# The Standardization and Quantification of Nuclear Factor Kappa B p65 by Real-Time Quantitative Polymerase Chain Reaction

**DOI:** 10.7759/cureus.74715

**Published:** 2024-11-29

**Authors:** Sivasubramaniyan Gnanaskandan, Urmila Karunakaran, Padma Srikanth

**Affiliations:** 1 Microbiology, Sri Ramachandra Institute of Higher Education and Research, Chennai, IND; 2 Virology, Manipal Institute of Virology, Manipal, IND; 3 Microbiology, Retired-Private Practice, Chennai, IND

**Keywords:** hiv-1, nf-kb p65, peripheral blood mononuclear cells (pbmc), real-time quantitative pcr, standardization in research

## Abstract

The accurate quantification of nuclear factor Kappa B p65 (NF-κB p65) is critical for understanding inflammatory mechanisms, especially in HIV-1 infected individuals, where NF-κB p65 contributes to chronic immune activation. Conventional methods such as enzyme-linked immunosorbent assay (ELISA) and western blotting are limited in terms of sensitivity and reproducibility. This study aimed to devise a standardized real-time quantitative polymerase chain reaction (RT-qPCR) assay for NF-κB p65 using specifically designed primers and a probe.

The human NF-κB p65 sequence was obtained from the National Center for Biotechnology Information (NCBI) database, and specific primers and probes were designed using Primer3 v4.1.0 software, optimizing melting temperature and guanine-cytosine (GC) content to ensure stability. Standards were synthesized using the ImmuGenix cloning kit (ImmuGenix Biosciences Pvt. Ltd., Chennai, India) at an initial concentration of 2.8 × 10^10 ^copies/ml, followed by serial dilutions to achieve a range from 10^6 ^to 10^2 ^copies/ml. RT-qPCR for NF-κB p65 was performed using the TAKARA master mix (Takara Bio, Kusatsu, Japan). The assay precision was evaluated through intra- and inter-assay measurements, with coefficient of variation (CV%) thresholds set at <5% and <10%, respectively. NF-κB p65 levels were quantified and analyzed in HIV-1 infected individuals and uninfected healthy controls.

The RT-qPCR assay showed high intra-assay precision, with CV% values ranging from 0.07% to 0.2%, indicating minimal variability within individual runs. Inter-assay reproducibility showed CV% values between 0.6% and 3.6%, confirming consistent performance across experimental runs. The cycle threshold (Ct) values for NF-κB p65 were lower among HIV-1-infected individuals, indicating higher expression compared to uninfected healthy controls.

Based on our findings, our standardized RT-qPCR protocol provides a reliable and reproducible approach for quantifying NF-κB p65, aiding in the understanding of inflammatory responses.

## Introduction

Nuclear factor kappa B (NF-κB) is a transcription protein that is essential for various physiological processes such as immune response, inflammation, cell survival, and apoptosis [1,2]. The NF-κB family comprises five subunits: p50, p52, Rel A (p65), c-Rel, and Rel-B. Rel A (p65) is the most significant among these, as it controls the expression of a diverse range of genes integral to inflammatory responses and immune regulation. NF-κB p65 usually resides in the cytoplasm, and, upon activation, it translocates to the nucleus, where it binds to the specific DNA sequences, which leads to the genes involved in cytokine production, immune cell signaling, and other critical immune functions [3,4]. NF-κB p65 has a crucial role in various pathological conditions such as chronic viral and bacterial infections, autoimmune disorders, and certain cancers that lead to persistent active inflammation [5,6]. Hence, it has a dual role in regulating both physiological and pathological processes.

NF-κB p65 is an intracellular protein, and quantifying NF- κB p65 levels in peripheral blood mononuclear cells (PBMC) provides essential insights into immune system activation and offers a valuable biomarker for health and diseases. PBMC is composed of lymphocyte and monocyte cells which serve to access the immune function, as they circulate in the bloodstream and reflect systemic immune activation [7]. The measurement of NF-κB p65 expression levels in PBMC cells helps to understand the inflammatory conditions and may also support clinical decision-making, particularly in diseases with persistent inflammation such as those related to HIV-1 and hepatitis B virus, as well as various cancers. Therefore, developing precise and reliable methods to measure NF-κB p65 is crucial for advancing research and clinical applications.

Conventional serological assays, such as enzyme-linked immunosorbent assay (ELISA), are effective for detecting extracellular antibodies in biological samples. However, their utility is limited for intracellular proteins like NF-κB p65, which is predominantly localized within the cytoplasm and nucleus. ELISA-based approaches lack the resolution to provide accurate measurements of intracellular protein expression, especially in the context of dynamic cellular processes such as inflammation. Western blotting, a gold-standard technique, is widely used for detecting and quantifying intracellular proteins. While this method offers specificity in identifying protein isoforms and post-translational modifications, it primarily provides data on protein abundance and not transcriptional activity. Hence, it is insufficient for elucidating the transcriptional regulation of NF-κB p65, which plays a key role in orchestrating inflammatory responses. These existing methodologies have certain disadvantages in evaluating NF-κB p65's functional activity during inflammation, particularly at the transcriptional level. Accurate quantification of NF-κB p65 mRNA expression is essential for understanding its role in inflammatory pathways and immune regulation. 

Real-time quantitative polymerase chain reaction (RT-qPCR) is the gold-standard technique for gene quantification, known for its high sensitivity, specificity, and wide dynamic range [8,9]. RT-qPCR is essential in both research and routine clinical laboratory practices for analyzing gene expression, specifically in studies focusing on complex immune responses. However, maintaining consistency in RT-qPCR is a major challenge due to variations in the primer and probe selection and the lack of standardized protocols. Such variability can have a serious impact on the reproducibility and comparability of the results, potentially limiting the broader application of findings across different research settings.

This study aims to address this critical gap by developing a standardized and reproducible RT-qPCR-based method to quantify NF-κB p65 mRNA levels, to provide a more precise tool for investigating its regulatory mechanisms and potential as a biomarker in inflammatory and immune-mediated diseases. We endeavored to devise a standardized real-time qPCR assay for NF-κB p65 by using specifically designed primers and a probe.

## Technical report

Methodology

In this study, we designed custom primers and probes specifically targeting NF-κB p65, along with synthesized standards for RT-qPCR. Then, the designed primers, probes, and standards were used to quantify and analyze the expression levels of NF-κB p65 in HIV-1 infected individuals and healthy controls.

Participant Recruitment

In this exploratory study, we aimed to quantify the NF-κB p65 levels in blood samples from 10 individuals infected with HIV-1 and 10 uninfected healthy controls using our designed primers, probes, and standards. Adult men aged between 18 and 65 years were included in the study. Participants with a medical history of surgery, diarrhea, antibiotic use, cancer, obesity, or any other medical condition and personal habits such as alcohol and tobacco use and intravenous drug abuse, were excluded from the study. Informed written consent was obtained from all the participants during recruitment.

Sample Collection

A total of 10 mL of fresh whole blood was collected from each participant into EDTA (ethylenediaminetetraacetic acid) vacutainers with the assistance of a phlebotomist, after obtaining informed consent. The samples were then moved to the laboratory within two hours under cold chain conditions between 2-8 ^o^C. 

PBMC Separation and mRNA Extraction

PBMCs were separated from the blood using the Ficoll-Hypaque method within six hours of collection as described in the literature [10]. mRNA was extracted from PBMCs using the RNeasy mini extraction kit (Qiagen, Hilden, Germany) as per the manufacturer’s instructions.

cDNA Conversion

Complementary DNA (cDNA) was synthesized from extracted mRNA using the high-capacity cDNA reverse transcription kit (Invitrogen, Waltham, MA), utilizing random primers and reverse transcriptase enzyme. The master mix was prepared with 7 µL of 10x buffer, 7 µL of 50 mM magnesium chloride (MgCl₂), 2 µL of dNTPs (10 mM concentration), 1 µL of MMLV (Moloney murine leukemia virus) reverse transcriptase, 1 µL of random primers (2.5 µM concentration), and 12 µL of nuclease-free water, yielding a total reaction volume of 30 µL. Subsequently, 40 µL of the RNA template was added to this mixture. The thermal cycling conditions were as follows: an initial denaturation step at 97 °C for 10 minutes, followed by 40 cycles consisting of annealing at 37 °C for 60 seconds and extension at 95°C for five minutes. The DNA template was carried out with NF-κB p65 RT-qPCR.

Primer and Probe

The human NF-κB p65 sequence was retrieved from the National Centre for Biotechnology Information (NCBI) database to design the specific primers and a probe. Primer and probe design was performed using Primer3 v4.1.0 online software. During primer design, the melting temperature (Tm) was optimized at 60 °C to promote stable binding. At the same time, the GC content was controlled within a 40-60% range to enhance structural stability and minimize secondary structure formation. Primer lengths were set between 20-25 base pairs (bp) to ensure high specificity and efficient amplification. The primers and probe were synthesized by ImmuGenix Bioscience Pvt. Ltd. (Chennai, India). NF-κB p65 mRNA generally has a short half-life (30 mins), typically ranging between one and four hours across various cell types [11]. According to the company, the primers were designed to be located closer to the 5’ end of the NF-κB p65 transcript, which is expected to enhance the detection rate even for transcripts with a short half-life.

Standards Preparation

The NF-κB p65 standards were synthesized by ImmuGenix Bioscience Pvt. Ltd. As per the company, the NF-κB p65 standards used in this study were prepared by synthesizing plasmids containing known concentrations of NF-κB p65 sequences using the IGB cloning kit (ImmuGenix Bioscience). These plasmid-based cloned standards were specifically designed for RT-qPCR to ensure accurate and reliable quantification of NF-κB p65 mRNA levels. The preparation methodology for these standards is proprietary to the company and has not been disclosed by the manufacturer. 

RT-qPCR Protocol

The NF-κB p65 standards provided by ImmuGenix had an initial concentration of 2.8 × 10^10 ^copies/ml. Ten-fold serial dilutions were performed using Milli-Q water to achieve final concentrations ranging from 10^6 ^to 10^2 ^copies/ml. The master mix was prepared with 5 μL of probe universal TAKARA master mix (Takara Bio, Kusatsu, Japan), 3.1 μL of nuclease-free water, 0.2 μL each of our specifically designed forward and reverse primers, 0.4 μL of the probe, 0.1 μL of ROX reagent, and 1 μL of the cDNA template, yielding a final volume of 10 μL, per sample. The cycling condition of RT-qPCR was as follows: initial denaturation at 95 °C for 30 seconds, followed by 35 cycles with denaturation at 95 °C for five seconds and annealing/extension at 60 °C for 30 seconds. Amplification and real-time fluorescence signals were monitored using the Rotor-Gene Q instrument, which produced an amplification curve. NF-κB p65 levels were then quantified by comparing the cycle threshold (Ct) values of each sample to a standard curve, constructed from the diluted standards, with GAPDH serving as the internal control gene. The primers and probes sequence details were not disclosed due to patent protection (patent number: 202441054401).

Statistical Analysis

To assess the efficiency and consistency of the designed primers and probes, a linear regression model was applied using R software version 4.3.2 to determine the R^2^ and p-values. PCR efficiency was calculated using the slope of the regression model by applying the following formula: Efficiency = 10(−1/slope) − 1 × 100 [12,13]. To evaluate repeatability (intra-assay variation), each NF-κB p65 RT-qPCR run included replicates of five randomly selected samples. The standards were prepared using 10-fold serial dilutions, with only the last four dilutions utilized for the assay. The coefficient of variation (CV%) for repeatability was analyzed based on Ct using the following formula: [CV = Standard Deviation (SD) of Ct/Mean Ct × 100]. To evaluate the reproducibility (inter-assay variations), two independent NF-κB p65 qPCR runs for five randomly selected samples were tested on different days. CV% for reproducibility was analyzed using the following formula [CV = Standard Deviation (SD) of Ct/Mean Ct × 100]. To identify the significant difference in NF-κB p65 levels from the PBMC of the HIV-1 infected individuals and healthy controls, a non-parametric Wilcoxon rank-sum test was conducted using Ct values.

Results

Efficiency of the Designed Primer and Probe

**Figure 1:**
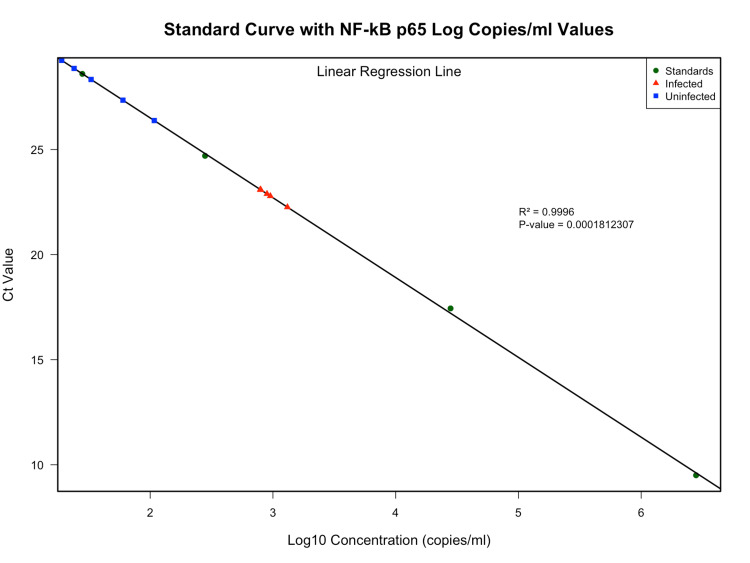
Standard curve plot for NF-κB p65 RT-qPCR assay Standard curve plot with log10 concentration of standards (copies/ml) on the X-axis and RT-qPCR Ct values on the Y-axis. A linear regression model was used to evaluate the efficiency of the standards (circles, green) by determining the R-squared (R² = 0.99) and p-value (<0.001), indicating strong correlation and statistical significance. HIV-1 infected participants (triangles, red) and uninfected participants (squares, blue) are also shown Ct: cycle threshold; HIV: human immunodeficiency virus; NF-κB p65: nuclear factor Kappa B p65; RT-qPCR: real-time quantitative polymerase chain reaction

The designed primers and probe for NF-κB p65 based on the reference sequence from the NCBI database showed high levels of specificity and efficiency. The standards were diluted to 10-fold serial dilutions, with only the last four dilutions utilized. Assay performed across a wide range of concentrations of standards from a maximum of 2,80,000 copies/ml to a minimum of 1.250 copies/ml, yielded a robust curve with a correlation coefficient (R^2^) of 0.999 (Figure 1), reflecting a near-linear relationship between the plasmid DNA concentration and the Ct values, which obtained 98% efficiency derived from the standard curve slope. The elevated R^2^ value indicates that the NF-κB p65 detection was consistent and accurate across various concentrations, affirming the assay’s quantitative reliability.

Repeatability and Reproducibility of the Assay

**Figure 2:**
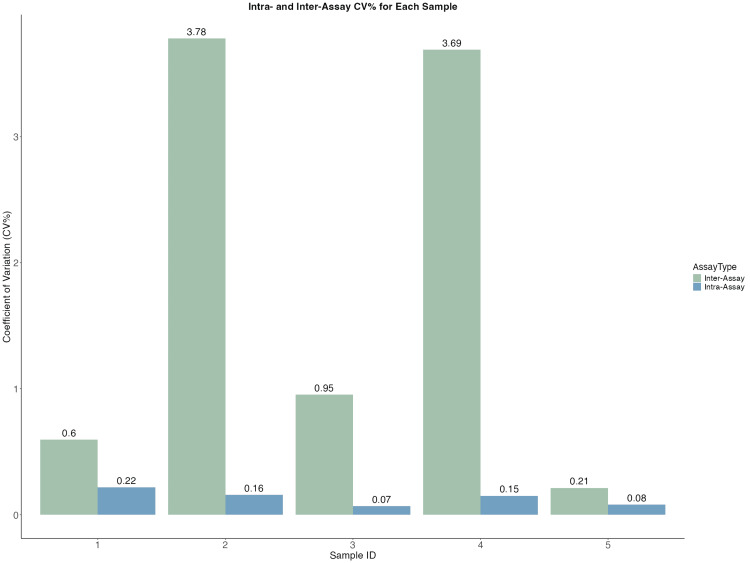
Coefficient of variation (%) of intra- and inter-assay variability Bar plot showing the CV% on the Y-axis and sample ID on the X-axis for intra-assay (mean CV%: 0.07%–0.22%) and inter-assay (mean CV%: 0.6%–3.6%) variability, demonstrating the high precision and reproducibility of the NF-κB p65 qPCR assay CV: coefficient of variation; NF-κB p65: nuclear factor Kappa B p65; RT-qPCR: real-time quantitative polymerase chain reaction

The intra-assay variability, which evaluates the consistency within a single experimental run, was assessed by calculating the CV% across replicates of randomly selected five samples from each group. Assay showed the mean intra-assay CV% ranging from 0.07% to 0.22% (Figure 2). Inter-assay variation is essential to confirm whether the standardized protocol can reliably produce comparable results with minor differences between two independent assays. The CV% between two independent runs of inter-assay ranges from 0.6% to 3.6% (Figure 2).

Sensitivity and Limit of Detection

**Figure 3:**
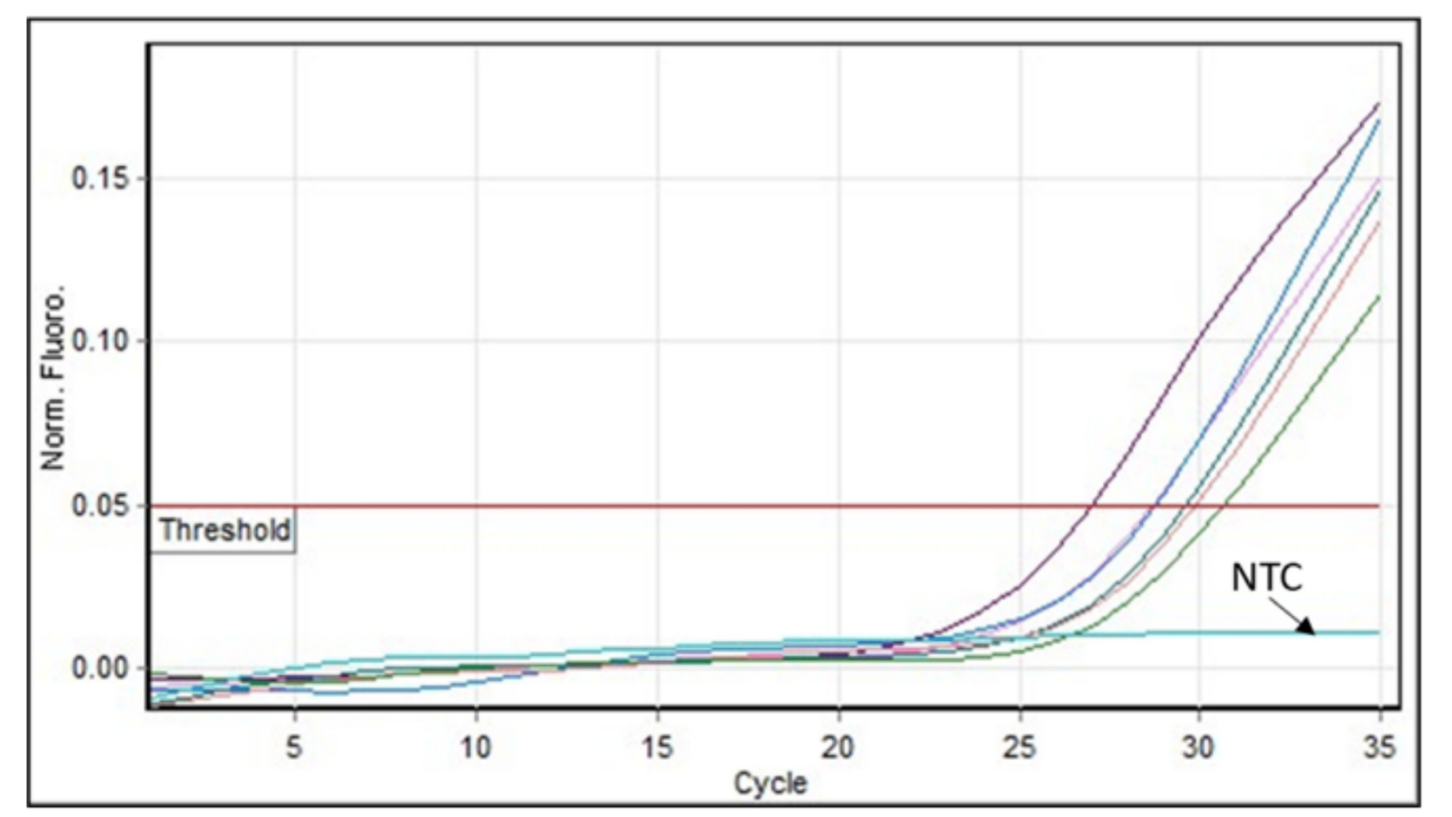
Amplification graph for LOD RT-qPCR amplification graph demonstrating the LOD for NF-κB p65 with diluted standards at various concentrations along with the non-template control showing no amplification. The dilutions used were smaller, around two-fold, to better determine the LOD LOD: limit of detection; NF-κB p65: nuclear factor Kappa B p65; RT-qPCR: real-time quantitative polymerase chain reaction

The limit of detection (LOD) was established by performing serial dilutions of the synthesized standards to identify the lowest concentration at which NF-κB p65 could reliably be detected. The dilutions used were smaller, around two-fold, to better determine LOD. These smaller dilutions allowed for more precise LOD determination, as shown in Table 1. The lowest level of NF-κB p65 detected by our assay was 1.228 copies/ml (Figure 3, Table 1), which indicates that the assay was capable of detecting even minimal levels of NF-κB p65. Additionally, we included a non-template control (NTC) along with the standards to ensure that there was no contamination or non-specific amplification in our reaction. The NTC consistently showed no amplification, confirming the reliability of our assay.

**Table 1:** LOD using diluted standards The table provides the Ct values and corresponding calculated concentrations for each standard including non-template control. Given concentration refers to the known concentration of the standards provided, while "Calculated concentration" represents the expression level detected in the assay, as calculated based on the standard curve Ct: cycle threshold; LOD: limit of detection; NTC: non-template control

No	Name	Type	Ct	Given concentration (copies)	Calculated concentration (copies)
1	Standard 1	Standard	27.00	280.000	288.314
2	Standard 2	Standard	28.71	28.000	22.393
3	Standard 3	Standard	28.80	14.000	19.561
4	Standard 4	Standard	29.63	7.000	5.677
5	Standard 5	Standard	29.90	3.500	3.816
6	Standard 6	Standard	30.66	1.250	1.228
7	Non-template control	NTC			

NF-κB p65 Expression in HIV-1 Infected Individuals Versus Healthy Controls

**Figure 4:**
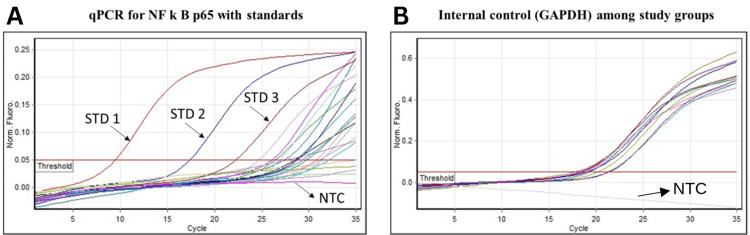
Quantification of NF-κB p65 among study participants (A) RT-qPCR amplification curve displaying normalized fluorescence (Norm. Flouro) on the Y-axis, indicating the increase in fluorescence signal during amplification. Ct values were determined from the fluorescence signals and compared to a standard curve generated using known concentrations of standards. (B) GAPDH was utilized as an internal control. No amplification was observed in the NTC for either NF-κB p65 or GAPDH Ct: cycle threshold; NF-κB p65: nuclear factor Kappa B p65; NTC: non-template control; RT-qPCR: real-time quantitative polymerase chain reaction

**Figure 5:**
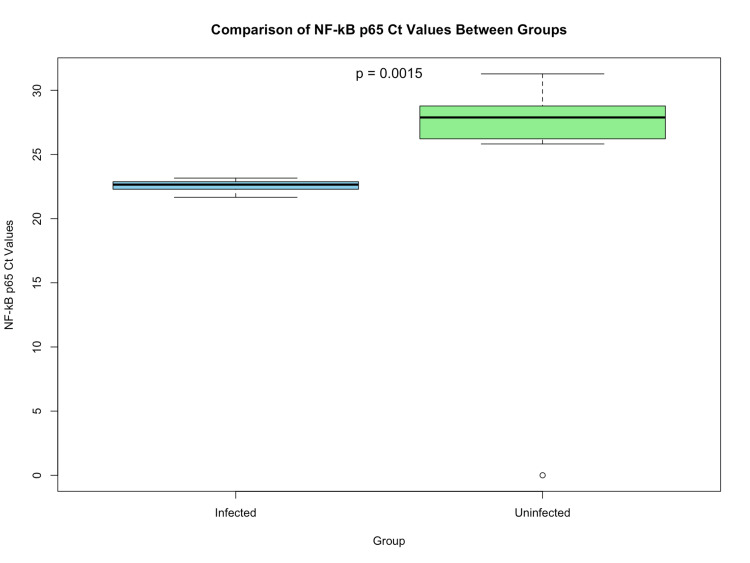
Comparison of NF-κB p65 cycle threshold values between HIV-1 infected individuals and healthy controls Box and whisker plot comparing the Ct values of NF-κB p65 expression between HIV-1 infected and uninfected individuals. The Y-axis represents Ct values, while the X-axis represents the groups (infected vs. uninfected). A Wilcoxon rank-sum test indicated statistical significance with p<0.01 Ct: cycle threshold; HIV: human immunodeficiency virus; NF-κB p65: nuclear factor Kappa B p65

HIV-1 infection is associated with persistent inflammation even if the individuals are on anti-retroviral therapy (ART). Hence, as an exploratory study, we included 10 HIV-1 infected and 10 uninfected people who are on ART and virally suppressed, to analyze the NF-κB p65 expression level (Figure 4A) along with internal control gene GAPTH (Figure 4B). The HIV-1 infected group had lower mean Ct values, indicating higher NF-κB p65 expression levels when compared to the uninfected group. Wilcoxon rank-sum test showed significant differences in Ct values between infected and uninfected groups (p<0.01) (Figure 5). The median [interquartile range (IQR)] of NF-κB p65 levels in HIV-1 infected participants were 723 (237) copies/ml, and those of uninfected healthy controls were 26.5 (41.75) copies/ml. These findings suggest that NF-κB p65 transcription is up-regulated in response to HIV-1 infection, which may reflect an immune activation state related to the infection.

## Discussion

RT-qPCR is a widely adopted diagnostic technique, and it is highly valued for its rapid processing, high sensitivity and specificity, and lower contamination risk relative to conventional diagnostic methods [14,15]. The RT-qPCR assay developed in this study offers clear advantages over traditional methods for measuring NF-κB p65. Conventional techniques such as ELISA, western blotting, chromatin immunoprecipitation (ChIP), and luciferase reporter assays have certain limitations.

ELISA, for instance, depends on antibody specificity, which can vary and potentially affect target detection accuracy since NF-κB p65 is an intracellular protein. Although western blotting is effective for visualizing protein expression patterns, it often lacks the quantitative sensitivity and precision offered by PCR-based methods [16]. ChIP, while valuable for analyzing protein-DNA interactions, generally requires larger sample volumes and relies on antibody quality, which can impact assay specificity [17]. Moreover, the ChIP technique is labor-intensive and prone to variability, potentially limiting its quantitative accuracy and reproducibility. Similarly, the luciferase reporter assays measure transcriptional activity, and they can be influenced by cell conditions and depend on artificial constructs, which may not accurately reflect natural cellular processes [18].

The RT-qPCR assay optimized in this study exhibits high sensitivity and precision. The accepted optimal range of RT-qPCR assay’s efficiency is considered to be between 90-110%. Conditions such as poor primer binding or probe instability often impact the efficiency of the assay, reflecting values of 90% or above 110%, leading to inconsistent Ct values and unreliable quantification [19]. The 98% efficiency of the assay highlights that the designed primer and probe can facilitate exponential amplification with minimal variation, thereby ensuring reliable target detection. Also, the observed 98% efficiency validates the robustness of the assay, as each PCR cycle approximately doubles the target DNA, demonstrating sensitivity and specificity for the quantification of NF-κB p65.

The CV% for intra-assay (repeatability) is generally considered acceptable below 5%. In our optimized RT-qPCR assay, intra-assay CV% values ranged from 0.07% to 0.2%, demonstrating robust and consistent amplification within individual runs. This minimal variability underscores the precision and reliability of the assay in quantifying NF-κB p65 levels without significant fluctuations within a single experiment. Inter-assay reproducibility further confirmed the assay’s robustness, with CV% values ranging from 0.6% to 3.6%, well below the commonly accepted 10% threshold for reliable RT-qPCR assays. These results indicate that the assay provides highly consistent and precise quantification of NF-κB p65 expression across multiple runs and over time.

The primer design process involved optimizing the melting temperature to 60 °C and the GC content to 40-60%, which helped stabilize the primers and prevent the formation of undesirable secondary structures like hairpins or dimers that could interfere with accurate amplification [20]. Additionally, the primers were designed to be 20-25 nucleotides in length, ensuring specificity for the NF-κB p65 target region and compatibility with RT-qPCR conditions. The validated primer-probe design enables accurate quantification of NF-κB p65 expression, capturing low levels of NF-κB p65 (LOD - 1.22 copies/ml) with minimal variability. The assay demonstrated minimal non-specific amplification, validating it as a precise and robust tool for quantifying NF-κB p65, suitable for both research and clinical applications.

The standardized RT-qPCR protocol for quantifying NF-κB p65 expression has a wide range of clinical and research applications, particularly for monitoring diseases characterized by NF-κB activation, such as HIV-1 infection, chronic inflammatory conditions, and cancers. In this study, NF-κB p65 levels were higher in HIV-1 infected individuals compared to healthy controls, even with a small sample size. This suggests that, clinically, this assay could serve as a sensitive biomarker tool for assessing immune activation levels, potentially enhancing the evaluation of disease progression and treatment efficacy. Given the link between elevated NF-κB p65 expression and chronic inflammation or immune dysregulation in conditions like HIV-1 and autoimmune disorders, this assay provides valuable insights into patient responses to anti-inflammatory or antiretroviral therapies.

Limitations

The main objective of this study was to standardize the quantification of NF-κB p65 from PBMCs using RT-qPCR with specifically designed primers and a probe. Given the exploratory nature of this study, the sample size was limited to 20 participants, which is relatively small.

Strengths

This study established a reliable method for quantifying NF-κB p65 expression using RT-qPCR with custom-designed primers and a probe. The method demonstrated high sensitivity and reproducibility, providing a strong foundation for its application in future studies with larger sample sizes and diverse populations.

## Conclusions

The RT-qPCR assay developed in this study demonstrates significant functionality in clinical and research applications focused on the role of NF-κB p65 in immune response and inflammatory processes, particularly in HIV-1 infection. The assay provides a reliable method for quantifying and evaluating NF-κB p65 expression as a potential biomarker for immune activation. Future studies should build on this work by investigating NF-κB p65 expression in larger cohorts and performing comparative analyses with established molecular and serological assays to validate our findings.
